# MMP-13 enzyme and pH responsive theranostic nanoplatform for osteoarthritis

**DOI:** 10.1186/s12951-020-00666-7

**Published:** 2020-08-27

**Authors:** Qiumei Lan, Rongbin Lu, Haimin Chen, Yunfen Pang, Feng Xiong, Chong Shen, Zainen Qin, Li Zheng, Guojie Xu, Jinmin Zhao

**Affiliations:** 1 Guangxi Engineering Center in Biomedical Materials for Tissue and Organ Regeneration, The First Affiliated Hospital of Guangxi Medical University, Nanning, 530021 China; 2 Research Centre for Regenerative Medicine, Department of Orthopedics, The First Affiliated Hospital of Gaungxi Medical University, Nanning, 530021 China; 3 Guangxi Collaborative Innovation Center for Biomedicine, The First Affiliated Hospital of Guangxi Medical University, Nanning, 530021 China; 4 Department of Orthopaedics Trauma and Hand Surgery, The First Affiliated Hospital of Guangxi Medical University, Nanning, 530021 China

**Keywords:** MMP-13/pH sensitive, Cartilage targeting, Osteoarthritis, Theranostics

## Abstract

Stimulus-responsive therapy permits precise control of therapeutic effect only at lesion of interest, which determines it a promising method for diagnosis and imaging-guided precision therapy. The acid environment and overexpressed matrix metalloproteinases-13 (MMP-13) are typical markers in osteoarthritis (OA), which enables the development of stimulus-responsive drug delivery system with high specificity for OA. We herein demonstrate a nano-micelle based stimuli-responsive theranostic strategy with reporting and drug release controlled by acidic pH and MMP-13 for OA therapy. Such nanoplatform is incorporated with a motif specifically targeting on cartilage, a motif responsive to matrix metalloproteinases-13 to specifically report OA condition and biodynamics of nano-micelles, an anti-inflammatory drug (e.g., psoralidin (PSO)) from traditional Chinese medicine, and a biocompatible polymeric skeleton for sustainable drug release in response to the acidic OA condition. The high effectiveness of this targeted precision therapy is demonstrated comprehensively by both in vitro and vivo evidences.

## Introduction

As the most common chronic joint disease, osteoarthritis (OA) is regarded as the primary cause of disability among older adults [1, 2]. Systemic administration and intra-articular injections of glucocorticoids or non-steroidal anti-inflammatory drugs (NSAID) are hard to exert maximize therapeutic benefit due to the avascular tissue on articular surface, the poor bioavailability and short half-life of drug [3], and rapid drug clearance [4]. Significant efforts have focused on engineering drug delivery systems to prolong the retention time of drug in the joint [5, 6]. In recent years, emerging stimulus-responsive smart systems for targeted imaging and precision therapy have attracted increasing interest, such as pH [7, 8], ROS [9] or NO [10] triggered drug release systems upon exposure to inflammation tissues, which not only prolonged drug release, but also increased specificity to tissue and cells. The selection of optimal stimulus for the smart drug delivery system is the key for OA therapy.

During the process of OA, the increase of active protease can degrade the cartilage ECM, which is considered as the main cause of cartilage destruction, such as matrix metalloproteinases (MMPs), adamalysin-like metalloproteinases with thrombospondin motifs (ADAMTSs), and cathepsins. Among them, matrix metallopeptidase 13 (MMP-13) that can cleave cartilage type II collagen is considered as one of the major therapeutic targets. MMP-13 is highly expressed in the hyaline cartilage of OA patients in comparison to healthy cartilage [11]. With the alteration in the physical environment and release of inflammatory mediators by articular cells (under disease conditions), an acidity in the OA environment is generated [9, 12]. The unique expressed MMP-13 [13, 14] and acidic pH [9] in OA joints enable the development of smart drug delivery system.

Poly (2-ethyl-2-oxazoline)-poly (ε-caprolactone) (PEOz-PCL or PPL) is a biocompatible and biodegradable polymer [15]. It has been utilized to construct pH-responsive drug carriers because protonation of the tertiary amine on PEOz backbone promotes endosome escape and drug release [16, 17]. Here, a specific collagen type II targeting peptide (Coll-II α1 chain-binding peptide–CollB) with the sequence of WRYGRL [18, 19] was grafted onto PPL to form cartilage targeting PPL (C-PPL). In parallel, PPL was conjugated with a specific peptide substrate of MMP-13 enzyme (H_2_N–GPLGVRGC–SH) [20, 21] labeled with a fluorescence dye (Cy5.5). Further, black hole quencher-3 (BHQ-3) that can quench Cy5.5 fluorescence was coupled via amide reaction to obtain MMP-13 responsive and pH sensitive polymer MR-Cy5.5-BHQ-3-PPL (MR-PPL). Lastly, cartilage-targeting and OA-specific theranostic nanoplatform (MRC-PPL) was obtained by self-assembly of C-PPL and MR-PPL, and was further employed as the carrier to load a traditional Chinese medicine, psoralidin (PSO), one of the active ingredients isolated from Psoralea corylifolia that has therapeutic effects on cardiovascular and inflammatory diseases [22, 23].

**Fig. 1:**
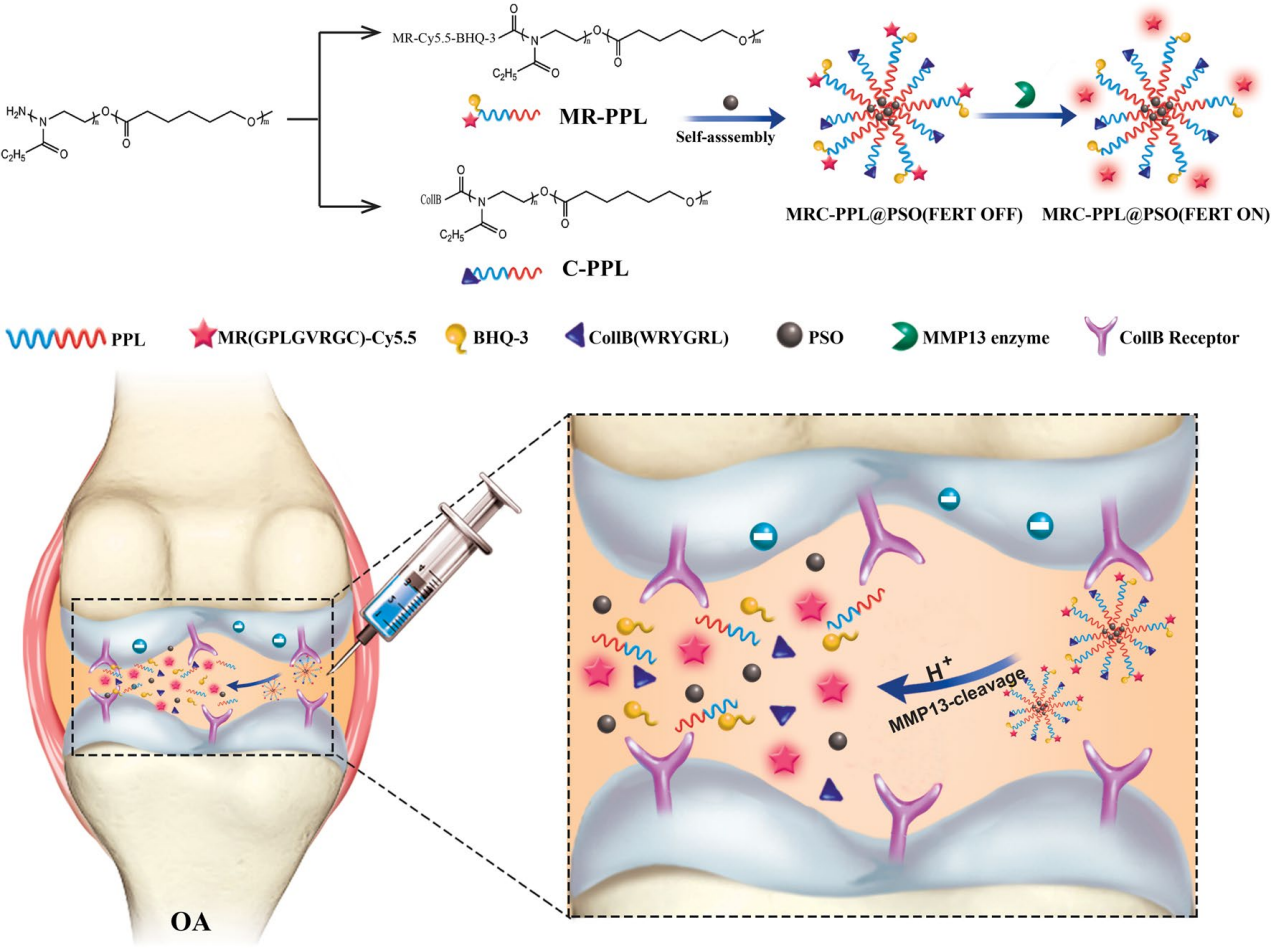
Schematic illustration of the synthesis and working mechanism of MMP-13 and pH responsive theranostic MRC-PPL@PSO nano-micelles for osteoarthritis

As schematically illustrated in Fig. 1, with intra-articular injection (IA) and guidance by CollB peptide, the multifunctional MRC-PPL@PSO nano-micelles specifically target on articular cartilage in the joint. Subsequently, abundant MMP-13 enzymes in OA environment cleave their substrate on the micelles, causing release of Cy5.5. The relief of quenching by BHQ-3 enables strong fluorescence signal from Cy5.5 whereby reporting the OA condition. Meanwhile, the acidic condition of OA leads to gradual disassembly of the micelles and consequent release of PSO molecules.

## Results

### Characterizations of MRC-PPL

**Fig. 2:**
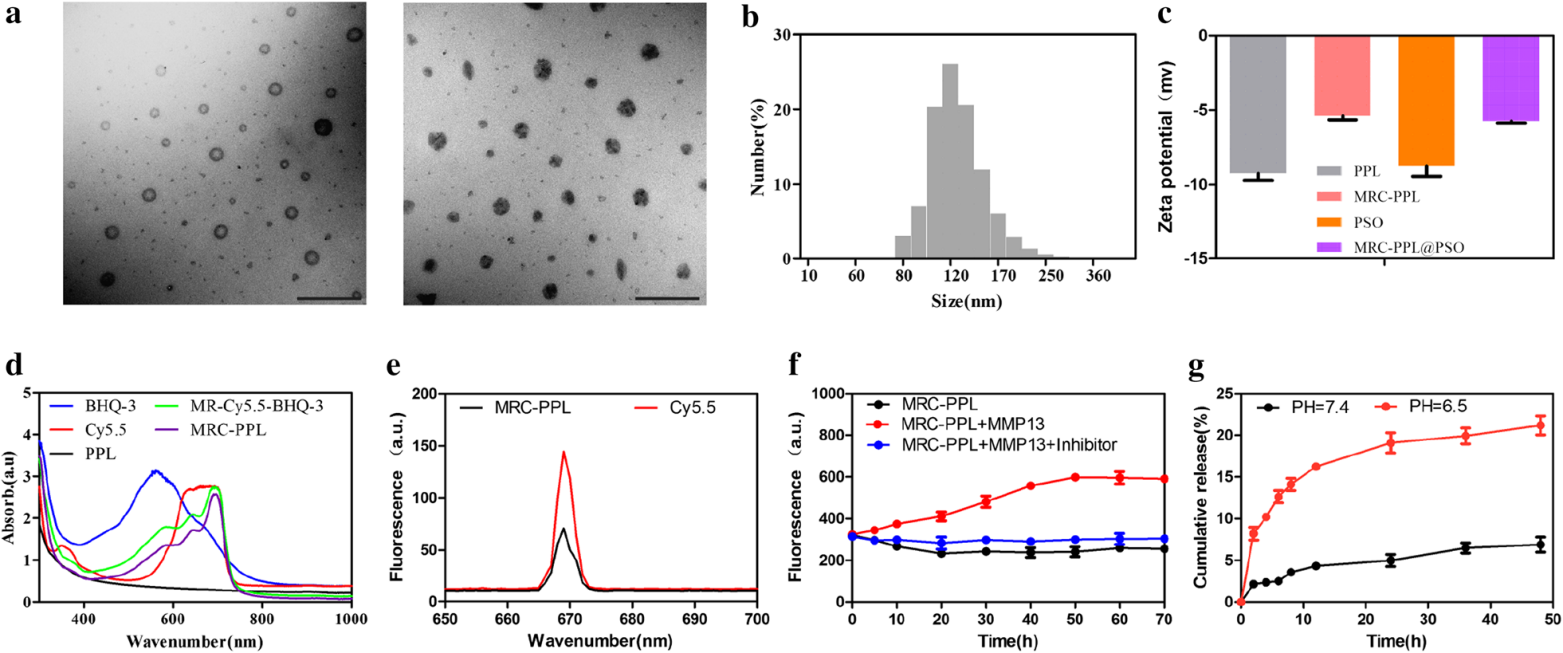
Characterization of MRC-PPL nano-micelles. **a** TEM images of MRC-PPL and MRC-PPL@PSO micelles. Scale bare = 500 nm. **b** Size distribution of MRC-PPL micelles based on dynamic light scattering. **c** Zeta potentials of PSO, PPL, MRC-PPL micelles and MRC-PPL@PSO micelles. **d** UV–Vis absorbance. **e** Fluorescence intensity of MRC-PPL micelles and Cy5.5. **f** Fluorescence intensity of MRC-PPL micelles, without or with MMP-13 (0.01 *μ*M), in the absence or presence of MMP-13 inhibitor (0.45 *μ*M). **g** *In vitro* release of PSO from MRC-PPL micelles in PBS (pH 6.5 and 7.4) with 0.1% Tween 80 (mean ± SD, n = 3)

PPL copolymers self-assembled to nano-micelles (120.3 ± 22.81 nm), as revealed by transmission electron microscopy (TEM, Fig. 2a). Similarly, the hydrodynamic diameter reported by dynamic light scattering (DLS) is 121.5 ± 26.1 nm (Fig. 2b). And loading micelles with PSO molecules using thin-film hydration method didn’t obviously alter the micelle size (Fig. 2a). Zeta-potential measurements (Fig. 2c) indicate that micelles consisting of only PPL are negatively charged (−9.25 mV), while MRC-PPL micelles are less negative (−5.38 mV). PSO is highly negatively charged (−8.75 mV) which hinders its cellular uptake since cell membrane is also negatively charged [19, 24]. Desirably, the negative charge of MRC-PPL@PSO micelles is significantly lesser (−5.75 mV). The successful construction of MRC-PPL was further confirmed by the blue shift induced by incorporation of BHQ-3 and Cy5.5 in MRC-PPL in UV–vis spectra (Fig. 2d).

Because of the energy resonance transfer to BHQ-3, the fluorescence intensity from Cy5.5 in MRC-PPL is much weaker than the equal amount of free Cy5.5 molecules (Fig. 2e). As demonstrated in Fig. 2f, introduction of MMP-13 enzyme to MRC-PPL dispersion led to gradual increases of fluorescence (becoming ~ 2 times brighter after 30 min), whereas the presence of MMP-13 inhibitor prevented this phenomenon. This observation confirms the MMP-13 specific (or OA-specific) fluorescence response, resulting from the cleavage of GPLGVRGC peptide in the probe and subsequent escape of Cy5.5 from quenching by BHQ-3.

### Drug release studies

The strong hydrophobicity of PSO has significantly prevented its application in OA. But through hydrophobic interaction it can be readily loaded into the core of MRC-PPL micelle via self-assembly. The loading percentage of PSO in the micelles is as high as 16.9%. The release of PSO from MRC-PPL@PSO at pH = 7.4 and pH = 6.5 was determined by high performance liquid chromatography (HPLC) in vitro. As shown in Fig. 2g, release of PSO was significantly accelerated by low pH (6.9% at pH 7.4 vs. 21.1% at pH 6.5 within 48 h). Evidently, MRC-PPL@PSO enables effective and sustained drug release under the acidic OA condition.

### In vitro cytotoxicity assessment

**Fig. 3:**
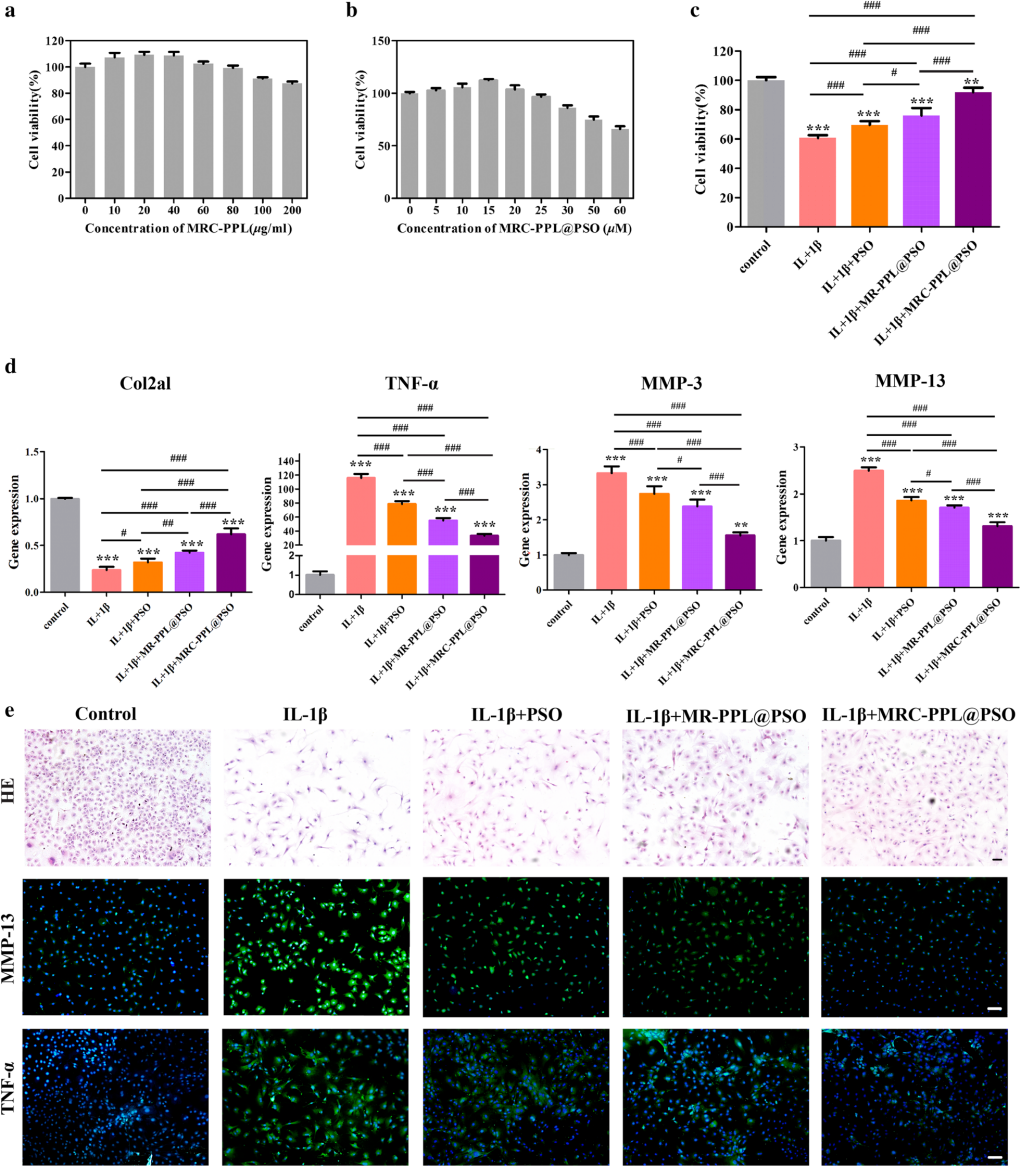
*In vitro* study on chondrocytes isolated from C57BL6/J mice. (**a** and **b**) Cell viability after treatment with MRC-PPL or MRC-PPL@PSO. (**c**) Cell viability after various treatments to IL-1β-stimulated chondrocytes. (**d**) Relative mRNA levels of Col2a1, TNF-α, MMP-3 and MMP-13 on IL-1β-stimulated chondrocytes with various treatments. (**e**) HE staining and immunofluorescence images. The nuclei were counterstained with DAPI (blue), and MMP-13 or TNF-α positive staining was stained with FITC (green). Scales bar: 400 *μ*m. Each data point represents mean ± s.d (n = 3). *, # indicate p < 0.05; **, ## indicate p < 0.01; ***, ### indicate p < 0.001

The cytotoxicity of micelles to chondrocytes isolated from C57BL6/J mice was investigated in vitro using CCK8 assay. Even at a high concentration (200 μg/mL) of MRC-PPL, the cell viability is  > 85% after 48 h of incubation, indicating its good biocompatibility (Fig. 3a). Then, the possible cytotoxicity of PSO-loaded MRC-PPL micelles was determined as the function of PSO concentration. Figure 3b shows that MRC-PPL@PSO micelles carrying 15 *μ*M PSO didn’t exert any cytotoxicity. Therefore, this dosage was chosen for the following experiments.

High level of interleukin 1 beta (IL-1β) is a marker for OA and is commonly used to induce inflammation of chondrocytes [25–27]. As showed in Fig. 3c, IL-1β stimulation (10 ng/mL) for 48 h caused 40% decrease of cell viability while MRC-PPL@PSO treatment largely rescued IL-1β induced cell death. We then used quantitative real-time PCR (qRT-PCR) to detect the gene expression levels of *TNF*-*α*, *MMP*-*3* and *MMP*-*13* with promoting cartilage destruction effect in each treatment group, as well as the chondroid-specific marker, *Col2a1*. As shown in Fig. 3d, the expressions of *TNF*-*α*, *MMP*-*3* and *MMP*-*13* are elevated by 115.72, 3.33 and 2.59 times respectively after IL-1β stimulation whereas the expression of Col2a1 was decreased to 23.78%. The upregulation of inflammatory factors and down-regulation of Col2a1 were significantly reversed by MRC-PPL@PSO treatment.

As shown in hematoxylin and eosin (HE) staining (Fig. 3e), chondrocytes with spindle shape and circular nucleate should be significantly transformed into elongated fibroblast-like cells after treatment of IL-1β for 24 h. MRC-PPL@PSO was able to restore the morphology of most IL-1β pre-treated chondrocytes. MMP-13 and TNF-α that play important roles in OA development were detected by immunofluorescence staining. As shown in Fig. 3e, positive staining in immunofluorescence staining (MMP-13 or TNF-α) in IL-1β-stimulated cells showed intense green fluorescence in IL-1β group. However, the fluorescence intensity was obviously decreased after treated by MRC-PPL@PSO.

Taking the observations in Fig. 3c–e together, we provide the in vitro evidence that MRC-PPL@PSO can efficiently rectify inflammation and rescue cartilage degradation in OA. PSO and MR-PPL @ PSO also play an anti-inflammatory role, but the effectiveness of PSO is much lower. MR-PPL@PSO micelles performed better than free PSO molecules because the engineered micelles can more effectively carry the hydrophobic drug into cells. MRC-PPL@PSO micelles outperformed MR-PPL@PSO micelles because it is equipped with cartilage targeting peptide.

### Anti-inflammatory effect of MRC-PPL@PSO on IL-1β-induced chondrocytes via the MAPK, NF-κB, and PI3K/Akt signaling pathways

**Fig. 4:**
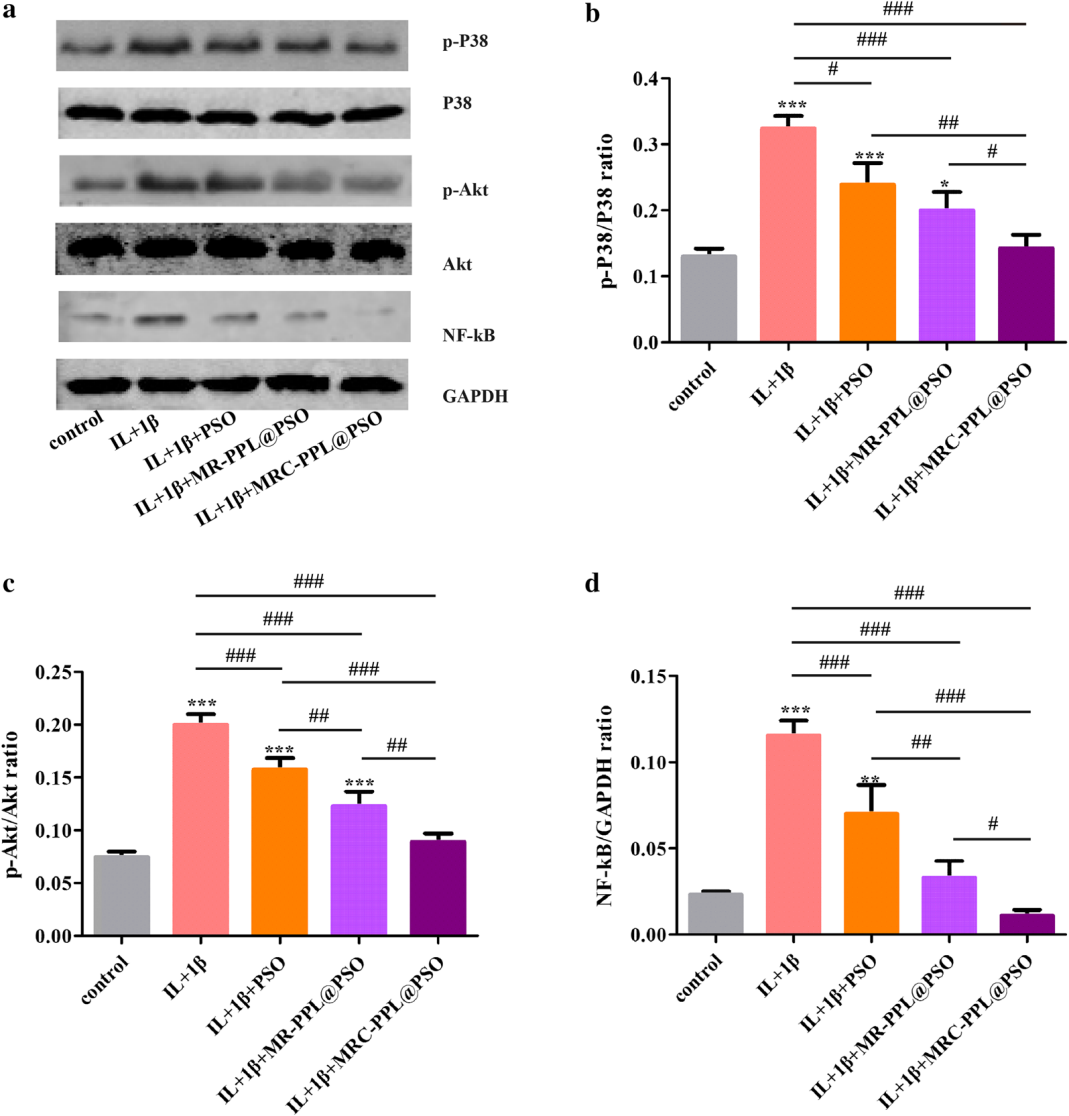
Molecular mechanism of MRC-PPL@PSO. The expression of PI3K/AKT, MAPK and NF-κB signaling pathway proteins p-P38, P38, p-Akt, Akt and NF-κB was determined by (**a**) western blot and (**c–d**) quantification analysis. Each data point represents mean ± s.d (n = 3). *, # indicate p < 0.05; **, ## indicate p < 0.01; ***, ### indicate p < 0.001

During the progression of osteoarthritis, IL-1β levels are elevated, which activates the nuclear factor NF-κB pathways to induce the expression of matrix metalloproteinases (MMPs) in cultured chondrocytes, leading to ECM degradation, abnormal bone metabolism and inflammatory disease [28, 29]. In addition, activated P38 MAPK has been reported to promote nuclear translocation of NF-κB [30]. Previous studies have found that PSO suppresses the expression of pro-inflammatory cytokines and chemokines as well as the expression of MMPs, which are the key regulators of cartilage destruction [22]. The PI3K/AKT signaling pathway also plays a crucial role in OA [31]. To explore the potential molecular mechanisms underlying the nano-platform treatment, the involvement of the MAPK, NF-κB and PI3K/Akt signaling pathways was investigated by Western blots. In vitro study showed that IL-1β-induced increase of phospho-P38/P38 (p-P38/P38), phospho-Akt/Akt (p-Akt/Akt) and NF-κB expression was diminished by each treatment group. (Figure 4a). Among all the treatment groups, MRC-PPL@PSO exhibited the greatest reduction of related proteins expression, superior to the PSO and MR-PPL@PSO groups. These results indicate that released PSO inhibits the production of inflammatory mediators in OA possibly mediated by the regulation of the PI3K/AKT pathway or MAPK cascades, leading to NF-κB inactivation.

### Cellular uptake of MRC-PPL

**Fig. 5:**
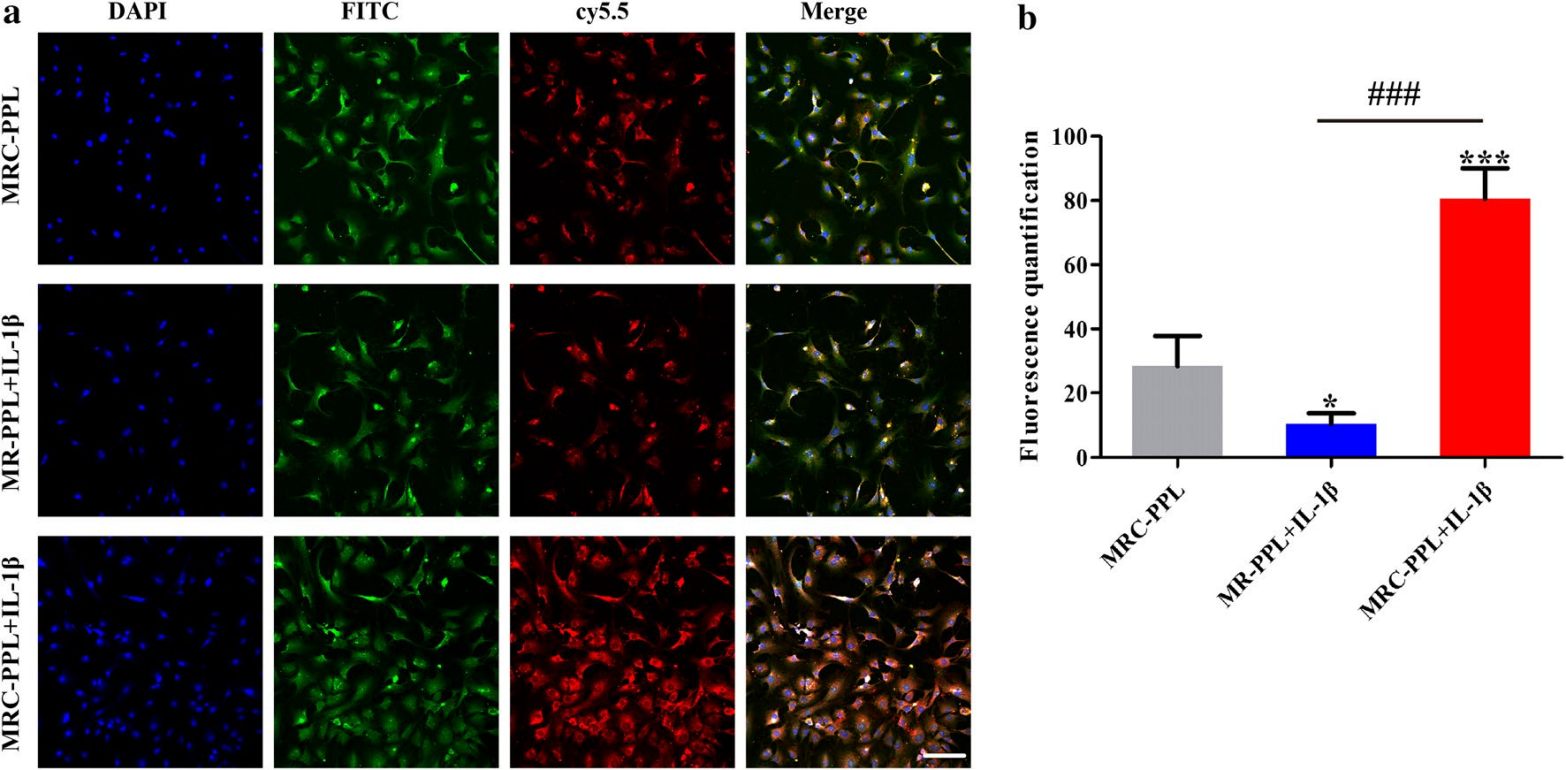
*In vitro* cellular uptake of MRC-PPL or MR-PPL micelles. **a** Immunofluorescence staining in chondrocytes to co-localized with collagen type II in the presence of MMP-13 or its inhibitor. The nuclei were counterstained with DAPI (blue) and collagen type II was stained with FITC (green). Scales bar: 400 *μ*m. **b** Fluorescence quantification of Cy5.5 after uptake of MRC-PPL or MR-PPL micelles by cells. Scale bars: 40 *µ*m. (n = 3; mean ± s.d; *, # indicate p < 0.05, **, ## indicate p < 0.01, ***, ### indicate p < 0.001.)

Cellular uptake and MMP-13 responsive behaviors of MRC-PPL micelles were investigated after 24 h incubation with normal chondrocytes and IL-1β stimulated chondrocytes. As shown in Fig. 5a, after administration of MRC-PPL, IL-1β stimulated cells exhibit much stronger fluorescence intensity (red) than normal chondrocytes cells, indicating that MRC-PPL micelles are efficient in response to excessive MMP-13 induced by IL-1β to release Cy5.5 due to the grafting of MMP-13 peptide substrate in MRC-PPL. Moreover, most red fluorescence emitted from Cy5.5 overlaps with the positive dye type II collagen (green fluorescence), suggesting MRC-PPL can successfully target to type II collagen produced by chondrocytes compared with MR-PPL (Fig. 5a). The results demonstrated that MRC-PPL is highly responsive to MMP-13 and can effectively target to type II collagen.

### Living imaging of animals

**Fig. 6:**
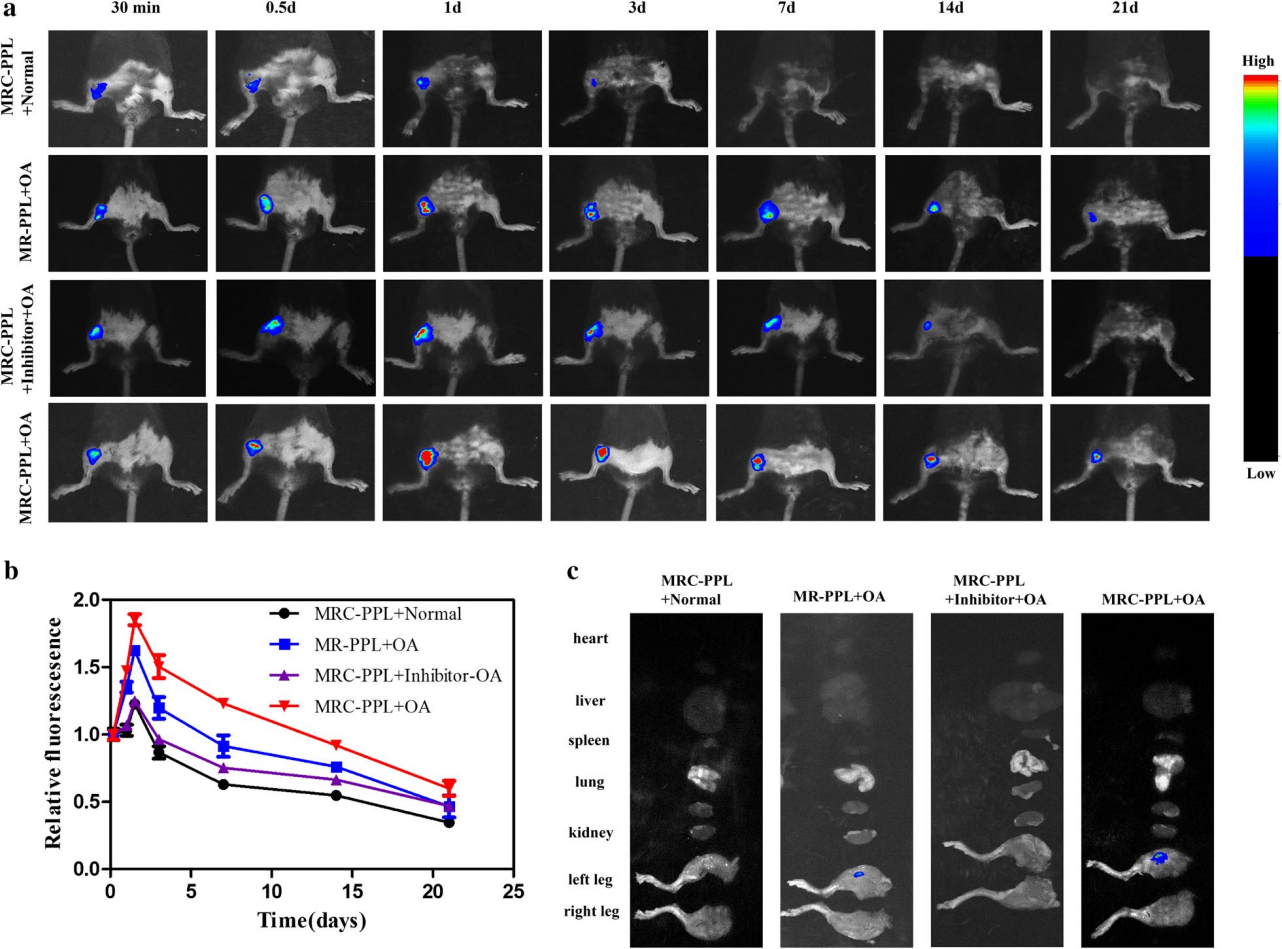
*In vivo* fluorescence imaging in normal or OA knees of mice. **a** Fluorescence imaging of OA joints injected with MRC-PPL, MRC-PPL + MMP-13 inhibitor, or MR-PPL at different time post-IA injection, as well as normal joint injected with MRC-PPL (excitation = 630 nm, emission = 700 nm). **b** The corresponding fluorescence intensity from OA joints at different times. **c** *Ex vivo* fluorescence imaging of heart, liver, spleen, lung, kidney, left knee and right knee at day 21 post-injection. (n = 5, mean ± S.D.)

Two weeks after IA injection of papain solution, OA developed in the knee joint of mice, showing mild swelling and deformity. As demonstrated by in vivo fluorescence imaging (Fig. 6a) and quantitative fluorescence intensity profile over time (Fig. 6b), MRC-PPL micelles had more accumulation and better retention in the OA joints than MR-PPL micelles, benefitting from the cartilage targeting peptide. In contrast, MRC-PPL micelles poorly accumulated in normal joints or OA joints or OA joints treated with MMP-13 inhibitor, due to insufficient presence of MMP-13 enzymes to activate the theranostic nanoplatforms. At day 21, fluorescence was still obvious in the MRC-PPL treated OA joint, but not in other organs including lung, spleen, heart, liver, kidney (Fig. 6c). The results of in vivo imaging are consistent with those of in vitro imaging. Taken together, we provide both in vitro and in vivo evidence that MRC-PPL micelles can specifically target on cartilage and label OA joint.

### MRC-PPL@PSO nano-micelles attenuate the progression of OA

**Fig. 7:**
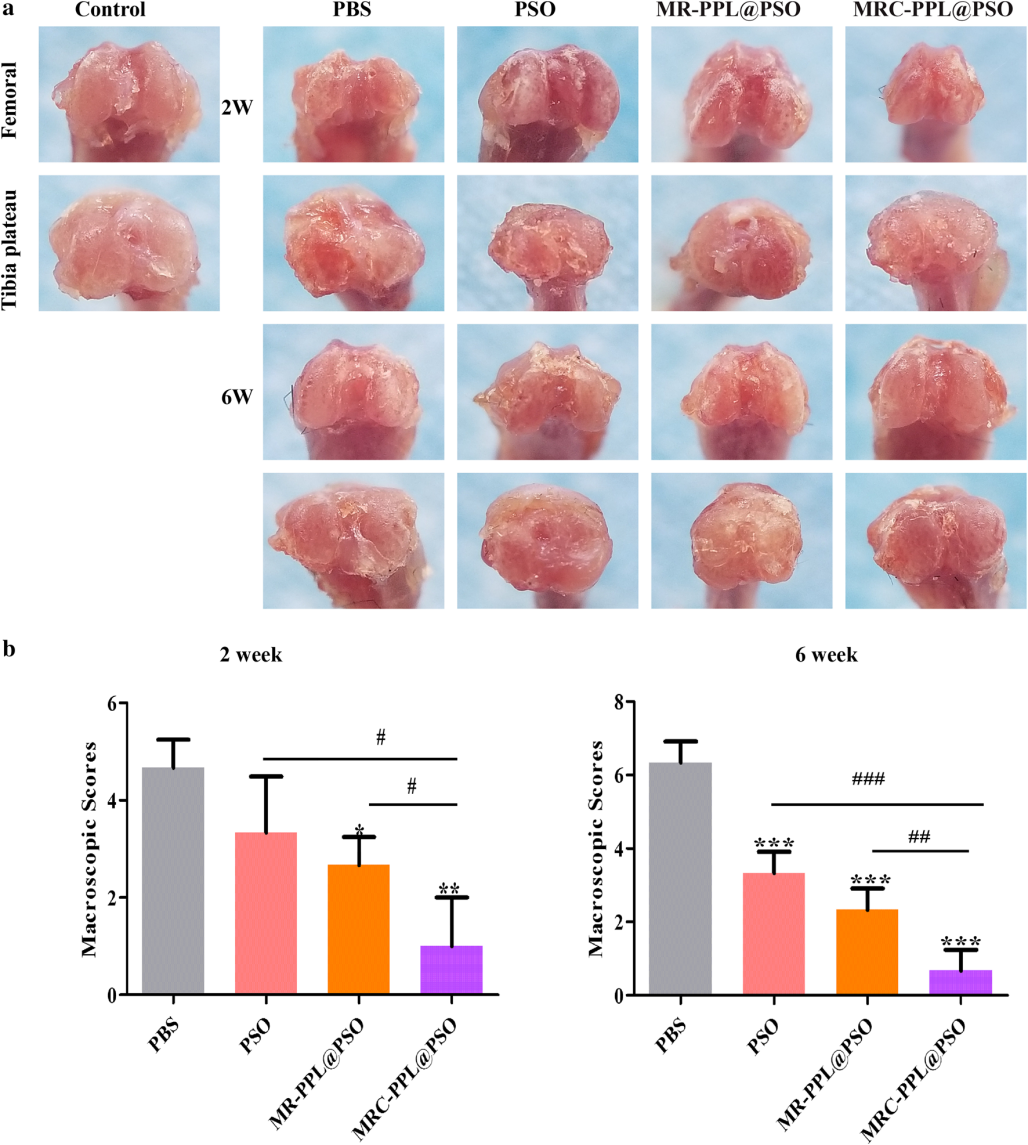
The macroscopic observation (**a**) and macroscopic score (**b**) of cartilage after IA-injection with PBS, PSO, MR-PPL@PSO and MRC-PPL@PSO for 2 and 6 weeks. Each data points represents mean ± S.D. (n = 5). *, # indicate p < 0.05; **, ## indicate p < 0.01; ***, ### indicate p < 0.001

After the mice receiving different treatments and being sacrificed at week 2 or 6, the femoral condyle and tibial plateau were collected and evaluated according to the criteria described by Lydia Wachsmuth et al. for the depth of erosion in articular cartilage [32]. Compared with the control (healthy) group, OA features represented by cartilage erosion and osteophyte formation and deterioration were found in PBS group (Fig. 7a). Osteophyte and surface lesion were significantly reduced at week 2 and 6 after administrating MRC-PPL@PSO, with macroscopic score reduction of 78.59% and 89.42% respectively (Fig. 7b). Although with much lesser degrees, PSO and MR-PPL@PSO were also able to reduce the scores by 47.38% and 63.19% respectively at week 6.

**Fig. 8:**
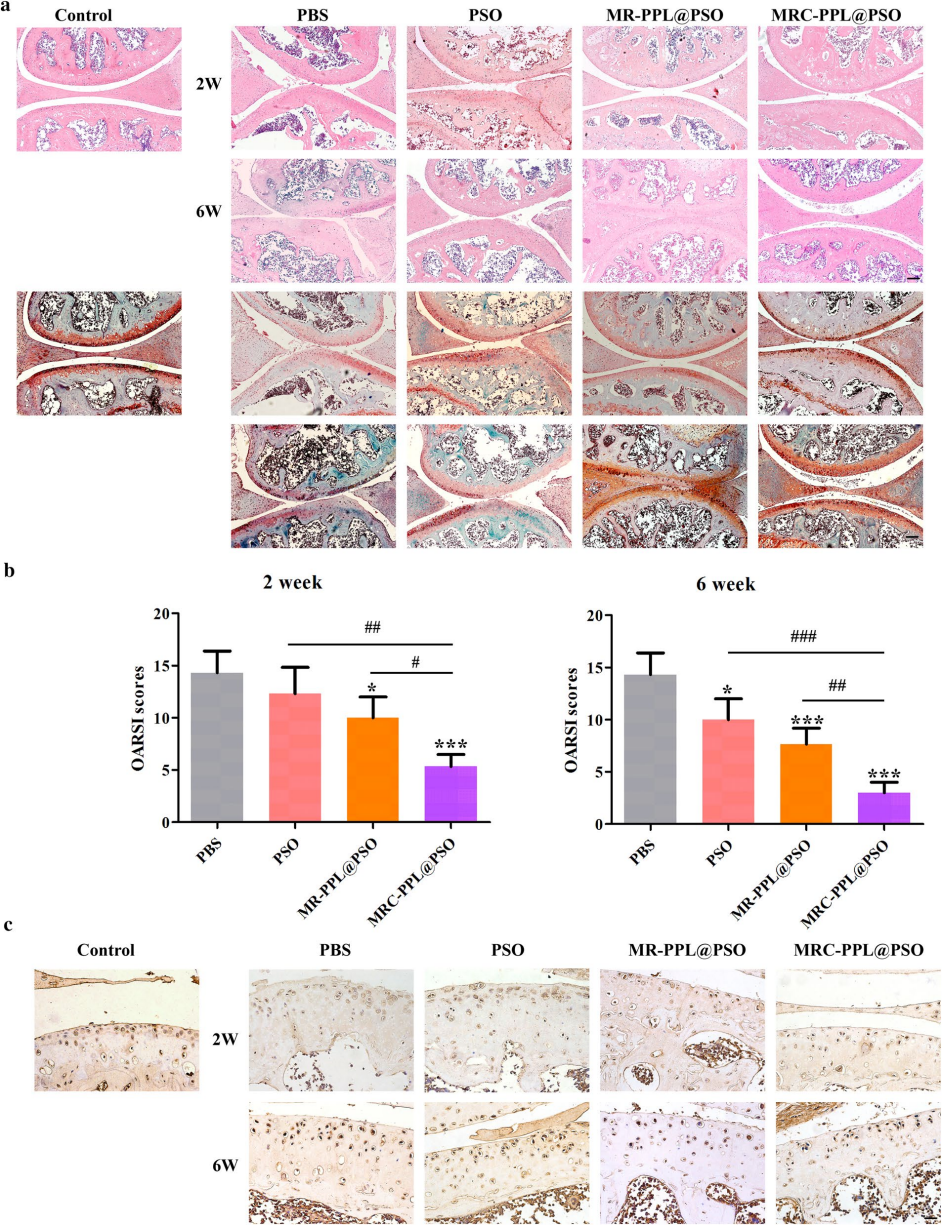
Histological analyses of different treatments for 2 or 6 weeks. **a** H&E (upper) and safranin-O/fast green staining (lower) of cartilage sections after the treatments with PBS, PSO, MR-PPL@PSO and MRC-PPL@PSO. Scale bar = 1 mm. **b** OARSI scores of articular cartilage after the treatments. Each data points represents mean ± S.D. (n = 5). *, # indicate p < 0.05; **, ## indicate p < 0.01; ***, ### indicate p < 0.001. **c** Immunohistochemical staining of MMP-13 on cartilage sections after the treatments. Scale bar = 300 *μ*m

The cartilage tissues were then evaluated by hematoxylin–eosin (H&E) staining and Safranin O-fast green staining. As shown in Fig. 8a, the cartilage layer with surface roughness, vertical cracks, erosion, denudation and deformation were observed in the PBS group, which were consistent with the characteristics of OA. Compared with the PBS group, all three treated groups showed different degrees of improvement in morphological change, matrix staining and tidemark integrity. Noteworthy, MRC-PPL@PSO can reduce the erosion and deformation of the cartilage layer surface, as well as the proliferation of tissue cells. Therefore, it is an effective method to maintain the columnar structure of cartilage. Furthermore, MRC-PPL@PSO group showed more intensive Safranin O staining (red) than other groups, indicating more secretion glycosaminoglycan (Fig. 8a). This phenomenon indicates that MRC-PPL@PSO facilitates deposition of glycosaminoglycan and attenuates cartilage matrix depletion and cartilage thinning. As presented in Fig. 8b, the OARSI scores based on histological analysis in all the treatment groups decreased to some extent compared with the PBS group, and the MRC-PPL@PSO group showed the lowest score with about 42.3% and 64.7% reduction at week 2 and 6, respectively. The protein expression level of MMP-13 was also assessed by immunohistochemistry staining in the cartilage at week 2 or 6. The MMP-13 positive staining in chondrocytes was observed to be dark brown in PBS group (Fig. 8c). On the contrary, the expression level of MMP-13 decreased after different treatments, following the order of PSO > MR-PPL@PSO > MRC-PPL@PSO. Noteworthy, the expression of MMP-13 in the MRC-PPL@PSO treated joints was nearly identical to the healthy control.

## Discussion

OA affected joints are presented by overexpressed MMP-13 and acidic microenvironment, which enables the development of ‘smart’ dual-stimuli-responsive probes for highly-efficient and controlled delivery and release of theranostic agents. Herein we demonstrated a theranostic nanoplatform for osteoarthritis, activated by microenvironment of acidic and MMP-13 enzyme overexpression in OA joints. The incorporated collagen type Il binding peptide facilitates the targeting and retention in the joint. Such disease specific stimulus-responsive strategy improves the efficiency and minimizes the side-effects. Upon activation, the theranostic nano-micelles (MRC-PPL@PSO) produce fluorescence signal and sustainably release the anti-inflammatory drug molecules. In IL-1β-treated chondrocytes, MRC-PPL@PSO promoted cell proliferation and inhibited inflammatory responses by down-regulating *TNF*-*α*, *MMP*-*3* and *MMP*-*13*, and significantly alleviated the cartilage lesions with the lowest OARSI score, as confirmed by histological staining and MMP-13 expression after 2 and 6 weeks post-treatment, indicating the protective and the targeting therapeutic effect on cartilage. Furthermore, MRC-PPL@PSO exerts antiarthritic effects by regulation of PI3K/AKT, MAPK and NF-κB signaling pathway.

In principle, multiple drug compounds can be loaded to achieve synergistic effects. In addition, such biocompatible PEOz-PCL based polymeric system can be readily modified for diagnosis and treatment of other diseases (e.g., cancers) by varying the targeting motif, enzyme substrates, and drugs.

## Materials and methods

### Materials

Poly(ε-caprolactone)-Poly(2-ethyl-2-oxazoline)-amine (PCL-PEOz-NH_2_, Mw 5000 Da) was purchased from Ruixibiotech (China). Psoralidin (PSO) and CCK8 were obtained from Sigma-Aldrich (US). Cy5.5-NHS and BHQ-3000S-5 was purchased from Seebio Biotech (China). N-Hydroxysuccinimide (NHS), N-(3-dimethylaminopropyl)-N’-ethylcarbodiimide hydrochloride (EDC•HCl) and N-succinimidyl-4-maleimide butyrate (GMBS) were supplied by J&K Scientific (China).

### Synthesis and characterizations of MRC-PPL micelles

MMP-13/pH responsive and cartilage targeting MRC-PPL micelles was obtained by self-assembly of C-PPL and MR-PPL [33]. Specifically, a Cy5.5 labeled MMP-13 substrate with the sequence of H_2_N–GPLGVRGC–SH was dissolved in DMSO (15 mmol, 500 μL) followed by the addition of N-succinimidyl-4-Maleimidobutyrate (30 mmol in 100 *μ*L DMSO) to form MR-Cy5.5. The reaction solution was incubated at room temperature for 4 h in a 1.5 mL eppendorf tube with gentle shaking. Then, to conjugate with the MMP-13 responsive peptide, EDC and NHS were added in MR-Cy5.5 solution, followed by the addition of 50 mg PPL. Subsequently, BHQ-3 was added in dark to form MR-Cy5.5-BHQ-3-PPL (MR-PPL), followed by the purification using HPLC. In parallel, WYRGRL peptide (10 mg in 100 μL DMSO) which specifically binds with collagen type II was coupled with PPL (50 mg in 100 μL DMSO) to obtain C-PPL. With addition of N, N-diisopropyl ethylamine (DIPEA, 10 μL) and overnight reaction, MRC-PPL micelles self-assembled from C-PPL and MR-PPL were finally obtained after ultrasonication and filtration through a 0.8-*μ*m membrane.

Polymeric micelles can encapsulate drug compounds using the film hydration method [34, 35]. The PPL copolymer (10 mg) and PSO (1 mg) were dissolved in acetonitrile and then evaporated in a vacuum at 50 °C until a dry film was formed. Subsequently, 2 mL ddH_2_O was added and vortexed. The morphology of MRC-PPL was uncovered by a transmission electron microscope (TEM, H-7650). We used Malvern Zetasizer Nano ZS90 to measure the size distribution and zeta potential of the micelles in aqueous suspension. Absorption spectra were obtained from a microplate reader (Thermo Scientific Multiskan GO Microplate Spectrophotometer). And the fluorescence from Cy5.5 was detected using a Fluorescence spectrophotometer (RF-5301PC).

The loading capability of PSO in MRC-PPL micelles was determined using the HPLC. Briefly, Lyophilized MRC-PPL@PSO powder was dissolved in HCl (0.1 mol/L) and moderate methanol was added to release PSO. After the supernatant was collected by centrifugation (6000 rpm, 30 min), the PSO concentration in the supernatant was detected by ACQ-BSM HPLC system (Waters, Milford, MA, USA).The system parameters were set to wavelength 347 nm, and the C18 analytical column (250 × 4.6 mm, 5 microns) was used at 37 °C. The mobile phase was consisted of methanol/water/0.05 M acetic acid (78/22/0.2, v/v) and the flow rate was 1.0 mL/min. The loading content (LC) of PSO were calculated according to the formula:

$$ \text{LC}\,\left( {\% } \right)\,\text{ = }\,\left( {\text{The}\,\text{mass}\,\text{of}\,\text{loaded}\,\text{PSO/The}\,\text{total}\,\text{mass}\,\text{of}\,\text{nanoparticles}} \right)\,{ \times }\,{10\% } $$

### Drug release studies

The pH-dependent release behavior of PSO from the MRC-PPL@PSO micelles was monitored at 37 °C. Briefly, 2 mL of the PSO-loaded micelles were placed in a dialysis bag (MWCO 3500), which was then immersed in PBS (10 mM, pH 6.5 and 7.4) containing 1% Tween 80. The release device was continuously shaken at 37 °C. Remove 1 mL of release media at predetermined intervals and immediately replace it with 1 mL of fresh media. The accumulative release amount of PSO was finally determined by HPLC.

### The chondrocytes extraction and culture

The chondrocytes was extracted from the knee joints of new born (3-5 days) C57BL6/J mice (Animal Resources Center of Guangxi Medical University, Nanning, Guangxi) after the articular cartilage were minced, digested with trypsin for 30 min, and digested with type II collagenase (2 mg/mL,Gibco). Chondrocytes were cultured in Eagle medium (Gibco) containing 1% (v/v) penicillin/streptomycin (Solarbio) and 10% (v/v) fetal bovine serum (Gibco). Cell experiments were constructed according to our previous work.

### Cytotoxicity test

The cytotoxicity of different formulations to chondrocytes cells were using Cell Counting Kit-8 (CCK-8; Dojindo Laboratories, Kumamoto, Japan). Briefly, the cell at a density of 5000 per well were seeded in 96-well plate overnight. Thereafter, MRC-PPL@PSO with the various amount of PSO was added and further incubated for 48 h. MRC-PPL micelles without PSO at different concentrations (0 ~ 200 μL/mL) were also tested for comparison. After treatment, 10 μL of CCK-8 were added to each well and cultivated for 4 h. Finally, the absorbance at 450 nm was measured using a microplate reader (Thermo Scientific Multiskan GO Microplate Spectrophotometer). The viability of MRC-PPL@PSO on the proliferation of IL-1β-induced chondrocytes was measured by CCK8 assay. Chondrocytes with density of 1.5 × 10^4^ cells/wells were cultured in 24-well plates, which were treated with 10 ng/mL IL-1β for 2 h to establish an in vitro model of osteoarthritis. Incubated with the PSO (15 µM), MR-PPL@PSO or MRC-PPL@PSO for 24 h. The latter two treatments equivalently contained 15 µM PSO. The remaining steps are based on the CCK8 method.

### In vitro cellular uptake

Cellular uptake of MRC-PPL was examined in chondrocytes. IL-1β (10 ng/mL) treated cells were added MRC-PPL or MR-PPL (1 mg/mL). Meanwhile, the cells were stained with immunofluorescence against COL2A1 (Boster, Wuhan, China, 1:200). After the nuclei were stained with DAPI, the images were captured using a laser scanning confocal microscope (Nikon A1, Tokyo, Japan). The excitation wavelengths for DAPI and Cy5.5 were 405 nm and 640 nm, respectively.

### In vitro anti-inflammatory activity and Immunofluorescence staining

The chondrocytes were seeded in 6-well (density of 5 × 10^4^ cells/cm^2^) or 24-well plates (density of 2 × 10^4^ cells/cm^2^). The cells pretreated with IL-1β (10 ng/mL) for 2 h were incubated with the PSO (15 µM), MR-PPL@PSO or MRC-PPL@PSO for 24 h. The latter two treatments equivalently contained 15 µM PSO. The cells were then washed with saline and fixed with 95% ethyl alcohol for 30 min, followed by staining with hematoxylin and eosin (HE, Nanjing Jian Biotechnology, China).The immunofluorescence was used to detect the protein expression level of MMP-13 and TNF-α. Samples were incubated with primary antibodies against MMP-13 (Boster, China; 1:100) and TNF-α (Boster, China; 1:100) at 37 °C for 4 h. The second antibody FITC-anti-rabbit IgG (Boster, China) was incubated in dark for 1 h. Finally, the nuclei were stained with DAPI, and the cells were observed with fluorescence microscope (Olympus, Japan).

### qRT-PCR detection

RNA separation kits (Megentec, China) were used to extract total RNA. The reverse transcription is done by a reverse transcription kit (Fermentas Company, USA). All qRT-PCR reactions were performed by a light Cycle 96 system (Roche, Switzerland) for 10 min at 95 °C, followed by 40 cycles with 10 s duration at 95 °C and then 60 s duration at 60 °C. The primers were used as follows in the Table 1.

**Table 1:** Primers for RT-PCR performance

Gene	Forward primer (5′-3′)	Reverse primer (5′-3′)	Size
TNF-α	CAGAAAGCATGATCCGCGAC	ATCCCTTTGGGGACCGATCA	20
MMP-13	TACCATCCTGCGACTCTTGC	TTCACCCACATCAGGCACTC	20
MMP-3	GTTCTGGGCTATACGAGGGC	TTCTTCACGGTTGCAGGGAG	20
Col2a1	ACACCGCTAACGTCCAGA TG	TCGGTACTCGATGACGGTCT	20
β-actin	CCCATCTATGAGGGTTACGC	TTTAATGTCACGCACGATTTC	20

### Western blot analysis

Proteins were extracted from chondrocytes with RIPA lysis buffer. Lysates were sonicated on ice and centrifuged at 12,000 rpm for 30 min at 4 °C. The protein concentration of the supernatant was determined using a BCA protein assay kit. Equal amounts of protein (40 μg) were separated by 10% SDS-PAGE and transferred to PVDF membranes. The membranes were incubated with blocking buffer 5% non-fat milk in TBS containing 0.1% Tween-20 (TBST) for 2 h at room temperature and then probed with the primary antibodies against p65, Akt and p-Akt, P38, p-P38 (dilution 1:1000) overnight at 4 °C. After washing three times with TBS containing 0.1% Tween-20 for 5 min, the membranes were then incubated with the secondary antibody (Invitrogen, USA) and visualized using the Odyssey Infrared Imaging System (LI-COR USA) according to the manufacturer’s instructions. Data were normalized by housekeeping protein (GAPDH).

### Induction of osteoarthritis in mice

A total of 100 C57BL/6 J mice (aged 6-10 weeks) were used for the in vivo experiments, which were performed in accordance with the ethical approval of the Institutional Ethics Committee of Guangxi Medical University. Arthritis was induced by articular injection of a mixture of papain solution (Sigma; 8% W/V) with l-cysteine (Sigma, 0.03 mol/L) three times a week. Mice were randomly divided into different experimental groups (n = 5): healthy control group, OA group and treatment groups. For OA group, mice were IA injected with 20 μL of PBS once per week. The treatments included IA injection of 20 μL PSO (15 µM), 20 μL MR-PPL@PSO solution (containing 15 µM PSO), or 20 μL MRC-PPL@PSO (containing 15 µM PSO), once per week for 2 or 6 weeks.

### In vivo near infrared fluorescence (NIRF) imaging

After anesthetized with 2% isoflurane (Aerrane; Baxter), mice were injected with treatment solution through the articular cavity (n = 5 for each group). In vivo fluorescence imaging was performed on an *IVIS* 200 system with a Cy5.5 filter set (excitation 630 nm; emission 695 nm). The mice were anesthetized and imaged at 30 min, 0.5, 1, 3, 7, 14, 21 d after injection.

### *Ex*-*vivo* NIRF imaging and histological analysis

At research endpoints, mice were euthanized and their hind knee joints were collected for fluorescence imaging by an IVIS-200 system. The treated articular cartilages were harvested. Grading of articular cartilage surface was performed by specifically evaluating a nine-area grid of each medial and lateral tibia plateau (scale of 0–8) [32]. Moreover, the treated joints were fixed with 4% formaldehyde for 48 h and decalcified with EDTA buffer. The samples were then embedded in paraffin and cut into sections of 3 microns. Paraffin sections were dewaxed and then collected for staining with hematoxylin (Solarbio, China). Glycosaminoglycan (GAG) secretion was detected using a safranin O-fast green kit (Solarbio, China) and MMP-13 secretion was assessed using a immunohistochemistry kit (Bioss, China). The stained slides were then imaged to examine the knee joint morphology on a fluorescence microscope. The OARSI cartilage OA histopathology grading system was adopted to evaluate the treated tissues [36].

### Statistical analysis

All data were reported as mean ± standard deviation of at least three experiments. The two-sided student t test was used for statistical analysis. P value less than or equal to 0.05 was considered statistically significant.

## Data Availability

The datasets used and/or analyzed during the current study are available from the corresponding author on reasonable request.
